# Combination epigenetic therapy in metastatic colorectal cancer (mCRC) with subcutaneous 5-azacitidine and entinostat: a phase 2 consortium/stand Up 2 cancer study

**DOI:** 10.18632/oncotarget.15108

**Published:** 2017-02-05

**Authors:** Nilofer S. Azad, Anthony el-Khoueiry, Jun Yin, Ann L. Oberg, Patrick Flynn, Douglas Adkins, Anup Sharma, Daniel J. Weisenberger, Thomas Brown, Prakriti Medvari, Peter A. Jones, Hariharan Easwaran, Ihab Kamel, Nathan Bahary, George Kim, Joel Picus, Henry C. Pitot, Charles Erlichman, Ross Donehower, Hui Shen, Peter W. Laird, Richard Piekarz, Stephen Baylin, Nita Ahuja

**Affiliations:** ^1^ Johns Hopkins University, Baltimore, MD, USA; ^2^ University of Southern California, Los Angeles, CA, USA; ^3^ Mayo Clinic, Rochestor, MN, USA; ^4^ Metro-Minnesota CCOP, Minneapolis, MN, USA; ^5^ Washington University, St. Louis, MO, USA; ^6^ Van Andel Research Institute, Grand Rapids, MI, USA; ^7^ University of Pittsburgh, Pittsburgh, PA, USA; ^8^ Mayo Clinic, Jacksonville, FL, USA; ^9^ Washington University, St. Louis, MO, USA; ^10^ Cancer Therapy Evaluation Program, National Cancer Institute, Bethesda, MD, USA

**Keywords:** epigenetics, colorectal cancer, DNA methyltransferases inhibitors, histone deacetylase inhibitors

## Abstract

**Purpose:**

Therapy with demethylating agent 5-azacitidine and histone deacetylase inhibitor entinostat shows synergistic re-expression of tumor-suppressor genes and growth inhibition in colorectal (CRC) cell lines and *in vivo* studies.

**Experimental Design:**

We conducted a phase II, multi-institutional study of the combination in metastatic CRC patients. Subcutaneous azacitidine was administered at 40 mg/m2 days 1-5 and 8-10 and entinostat was given 7 mg orally on days 3 and 10. An interim analysis indicated toxicity crossed the pre-specified safety boundary but was secondary to disease. A 2^nd^ cohort with added eligibility restrictions was accrued: prior therapies were limited to no more than 2 or 3 (KRAS-mutated and KRAS-wildtype cancers, respectively) and <30% of liver involvement. The primary endpoint was RECIST response. Serial biopsies were performed at baseline and after 2 cycles of therapy.

**Results:**

Forty-seven patients were enrolled (24:Cohort 1, 23:Cohort 2). Patients were heavily pre-treated (median prior therapies 4: Cohort 1 and 2.5: cohort 2). No responses were observed. Median progression-free survival was 1.9 months; overall survival was 5.6 and 8.3 months in Cohorts 1 and 2, respectively. Toxicity was tolerable and as expected. Unsupervised cluster analysis of serial tumor biopsies suggested greater DNA demethylation in patients with PFS above the median.

**Conclusion:**

In this first trial of CRC patients with combination epigenetic therapy, we show tolerable therapy without significant clinical activity as determined by RECIST responses. Reversal of hypermethylation was seen in a subset of patients and correlated with improved PFS.

## INTRODUCTION

Technological advancements in genome and epigenome science have led to an exponential increase in our understanding of cancer, but treatment avenues remain suboptimal. Promoter DNA hypermethylation can be correlated with inactivation of tumor suppressor genes, and has been increasingly recognized as an early and central event in carcinogenesis [1]. Such epigenetically silenced genes include *CDKN2A (p16)*, *MLH1, APC*, and the secreted frizzled related (*SFRP*) family and are associated with colon cancer [2–5]. Past work from our laboratories have confirmed that epigenetic changes are a frequent cause of decreased expression of these genes [6–8]. Our work has also shown that modulation of DNA methylation inhibits colon cancer formation in the Apc (*Min/+)* mice model [9]. Sequencing of the colorectal cancer (CRC) epigenome has revealed that these tumors contain hundreds of genes that are hypermethylated compared to normal tissue [7]. In addition, subsets of CRC patients have even greater hypermethylation of promoter regions, the CpG island hypermethylator phenotype or CIMP [10–11]. Importantly, epigenetic alterations in tumors show significantly more commonality between patients than do genetic mutations, also described by researchers in this group [7]. The complexity of the abnormal genome in solid tumors is the likely reason that molecularly targeted signal transduction inhibitors have had only modest efficacy in non-hematologic malignancies. Treatment designed to target abnormal DNA methylation in CRC might be a more powerful therapeutic strategy.

However, single-agent treatment in solid tumors has been unsuccessful. In cancer, there is substantial dysregulation of normal DNA methylation, with a global DNA hypomethylation of the CpG dinucleotides across the genome, concurrently with DNA hypermethylation of promoter associated CpG islands [1]. Epigenetic gene silencing due to aberrant DNA methylation is further mediated by the formation of repressive chromatin states due to changes in histone configuration [12]. Polycomb protein complexes affect chromatin configuration by interacting with repressive histone marks in the promoter regions of genes and with histone deacetylases (HDACs), resulting in gene repression through hypo-acetylation of histones [12]. These mechanisms can result in gene silencing due to a repressive chromatin configuration in the presence *and* absence of DNA methylation.

Both demethylating drugs and HDAC inhibitors have been approved in hematologic malignancies [13–15]. In solid tumors, single agent therapy with either treatment strategy results in limited tumor response, possibly due to high drug doses of drugs causing significant toxicity as well as the poor pharmacokinetics of these drugs with limited bioavailability [16–17]. Combination therapy of demethylating drugs along with HDAC inhibitors has been previously shown to promote gene re-expression [18]. We hypothesized that combination therapy with DNMT and HDAC inhibitors would be effective in reversing abnormal gene DNA methylation and thus resulting in therapeutic benefit in colorectal cancer patients. We designed a single arm phase II study to test this hypothesis in advanced colorectal cancer patients.

## RESULTS

### Candidate gene DNA methylation and expression assays

**Figure 1:**
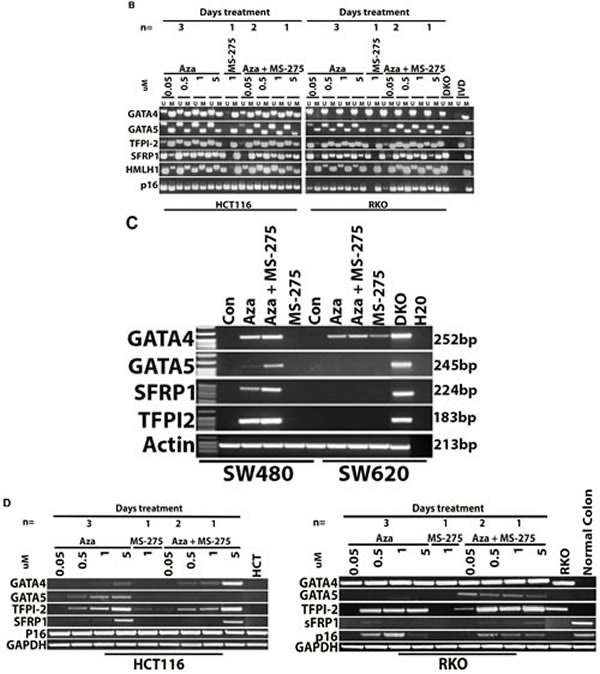
DNA methylation and gene expression changes with epigenetic therapy in cell lines **1A**. *and* **1B**. *MSP-based DNA methylation analysis.* Candidate tumor suppressor gene methylation of *GATA4*, *GATA5*, *TFPI2*, *SRFP1*, *MLH1* and *CDKN2A (p16)* was measured in four human CRC cell lines: SW480, SW620 HCT116, and RKO. U refers to unmethylated sequence, M indicates methylated sequence, while IVD and DKO are positive and negative controls for DNA methylation, respectively. Treatment with 5-azacitidine (AZA) caused demethylation of genes methylated at baseline across cell lines. However, no DNA methylation changes were evident after treatment with entinostat (MS-275). **1C**. *and* **1D.***mRNA expression upregulated with AZA and HDAC (MS-275) treatment.* Gel images show the mRNA expression levels as detected by semi-quantitative RT-PCR in the same cell lines. Cell lines (SW40, SW620, HCT116 and RKO) were treated with 5-azacitidine (AZA) and/or entinostat (MS-275). Treatment with entinostat alone did not result in expression changes of genes that are methylated at baseline, except for GATA4 expression in SW620 cells. Treatment with AZA resulted in increased expression of genes that are methylated at baseline, with additive effects seen in multiple genes after combining AZA and entinostat.

Human CRC cell lines were tested for DNA methylation status of selected candidate genes *GATA4, GATA5, SFRP1,* and *TPF12* and demonstrated baseline DNA methylation variability for each cell line. Treatment with azacitidine resulted in DNA demethylation (Figure 1A-1B) and increased expression of candidate genes if they were methylated at baseline and increased with addition of entinostat, also called MS-275 (Figure 1C-1D). Treatment with entinostat alone had no effect on DNA demethylation of the candidate genes nor on re-expression, except for *GATA4* in SW620 cells.

### Patient characteristics

From April 2010 to December 2011, forty-seven patients enrolled in the study; their demographic characteristics are presented in Table 1. Forty-five patients were evaluable for primary endpoint evaluation (23 patients in Cohort 1 and 22 patients in Cohort 2) after one patient in Cohort 1 was deemed ineligible after being treated with no baseline biopsy to confirm eligibility, and one patient in Cohort 2 withdrew before beginning treatment. Patients in both cohorts were heavily pretreated, with a median of 4 (range 2-9) prior therapies in Cohort 1 and 2.5 (range 2-6) prior therapies in Cohort 2. Most patients discontinued therapy due to disease progression (83.3% Cohort 1 and 81.8% Cohort 2); other reasons for study discontinuation were refusal of treatment (*n* = 3, 6.7%), adverse events (*n* = 4, 8.9%), and patient non-compliance (*n* = 1, 2.2%). The four adverse events accounting for study discontinuation reasons were recovery from surgery, grade 3 dyspnea and hypoxia, wound infection, and small bowel obstruction (all not related to study drugs).

**Table 1:** Demographics and patient information

	Cohort 1	Cohort 2
***N***	24	23
**Age Median (IQR)**	57 (28-75)	62.6 (32 - 75)
**Sex M:F**	13:11	14:9
**ECOG** ***N*** **(%)** **0** **1**	13(54.2%) 11(45.8%)	13 (56.5%) 10 (43.5%)
**Prior therapies Median (IQR)** **Previous oxaliplatin,** ***N*** **(%)** **Previous irinotecan,** ***N*** **(%)** **Previous EGF inhibitor,** ***N*** **(%)**	4.5 (2 - 9) 22 (95.7%) 20 (87.0%) 11 (47.8%)	3 (2 - 6) 20 (90.9%) 20 (90.9%) 4 (18.2%)
**Sites of disease** ***N*** **(%)** **Liver** **Lung** **Peritoneal** **Nodal** **Other**	18(75.0%) 19(79.2%) 8(33.3%) 20(83.3%) 9(37.5%)	18(78.3%) 17(73.9%) 12(52.2%) 10(43.5%) 6(26.1%)

### Treatment and dose reductions

Forty-six patients were started on the planned doses of 5-azacitidine and entinostat and were evaluable for toxicity endpoints. Median dose administered per treated cycle of azacitidine and entinostat across both cohorts was 100% (range 61-104% and 29-100%, respectively). Patients were treated for a median of 2 cycles in both cohorts (range 1-16).

### Treatment safety for both cohorts

Hematological and non-hematological toxicities in each cohort are shown in Table 2. In Cohort 1, the pre-specified toxicity boundary was crossed (3 patients out of the first 9 having grade 3 toxicities). Accrual was subsequently halted and a full toxicity analysis including review by the Data Safety Monitoring Board (DSMB) was performed. The toxicities responsible for crossing the toxicity boundary were grade 3 cellulitis (possibly related), grade 3 nausea (possibly related), and grade 3 hypophosphatemia (possibly related). It was determined by the DSMB that the toxicity being observed was most likely due to progression of disease in this very advanced cancer population rather than toxicity due to study treatment. The trial was amended to limit the number of prior therapies to no more than two (KRAS mutated patients) or three (KRAS wild-type patients including an epidermal growth factor receptor inhibitor) and disease burden to no more than 30% of the liver, in order to enroll patients that were more likely to be able to get a full two cycles of therapy without clinical deterioration. While the pre-specified threshold for toxicity was maintained for the new cohort, we excluded grade 3 fatigue, nausea, vomiting, abdominal pain, anorexia, and lymphopenia from the DSMB toxicity stopping rule based on the review of toxicity data in Cohort 1. These events are typically related to disease and not attributable to the study agents.

**Table 2:** Toxicities

	Cohort 1 (*N*= 24)				Cohort 2 (*N*= 22)			
Hematologic	Grade 1	Grade 2	Grade 3	Grade 4	Grade1	Grade 2	Grade 3	Grade 4
**Anemia**	4 (16.7%)	4 (16.7%)	3 (12.5%)		9 (40.9%)	3 (13.6%)		
**Leukopenia**	1 (4.2%)	1 (4.2%)			4 (18.2%)	2 (9.1%)	2 (9.1%)	
**Lymphopenia**	2 (8.3%)	2 (8.3%)	7 (29.2%)		2 (9.1%)	1 (4.5%)		
**Neutropenia**		1 (4.2%)			2 (9.1%)	1 (4.5%)	2 (9.1%)	
**Thrombocytopenia**	6 (25.0%)				4 (18.2%)	1 (4.5%)		
**Non-hematologic**								
**Abdominal pain**	2 (8.7%)				1 (4.5%)			
**Anorexia**	2 (8.3%)	2 (8.3%)	1 (4.2%)		3 (13.6%)	2 (9.1%)		
**Chest pain**							1 (4.5%)	
**Constipation**	1 (4.2%)	3 (12.5%)			2 (9.1%)	1 (4.5%)		
**Diarrhea**	2 (8.3%)	1 (4.2%)			1 (4.5%)			
**Fatigue**	5 (20.8 %)	6 (25.0%)	3 (13%)		8 (36.4%)	4 (18.2%)		
**Hypoglycemia**	3 (12.5%)	1 (4.2%)		1 (4.2%)	1 (4.5%)			
**Hypokalemia**	1 (4.2%)			1 (4.2%)				
**Hypocalcemia**	1 (4.2%)	1 (4.2%)			2 (9.1%)	1 (4.5%)		
**Hypophosphotemia**			2 (8.3%)			2 (9.1%)	1 (4.5%)	
**Hyponatremia**	3 (12.5%)		1 (4.2%)					
**Injection site reaction**	3 (12.5%)				3 (13.6%)			
**Nausea**	8 (33.3%)	8 (33.3%)	3 (12.5%)		6 (27.3%)	2 (9.1%)		
**Rash Maculo-papular**		1 (4.2%)			1 (4.5%)			
**Vomiting**	8 (33.3%)	3 (12.5%)	2 (8.3%)		4 (18.2%)			
**Dyspnea**	1 (4.2%)		1 (4.2%)		1 (4.5%)			
**Acidosis**			1 (4.2%)					
**Dehydration**		2 (8.3%)	1 (4.2%)					
**Urinary tract obstruction**							1 (4.5%)	

Toxicity was available for 46 patients that started treatment. Cohort 1 and Cohort 2 had similar overall toxicity profiles (Table 2). There were two grade 4 adverse events at least possibly related to study treatment in Cohort 1 (Hypoglycemia and Hypokalemia) and none reported for Cohort 2. The most frequently reported grade 3 drug-related toxicities were anemia (Cohort 1: 3 (12.5%); Cohort 2: 0), lymphopenia (Cohort 1: 7 (29.2%); Cohort 2: 0), neutropenia (Cohort 1: 0; Cohort 2: 2 (9.1%)), fatigue (Cohort 1: 3 (12.5%); Cohort 2: 0), hypophosphatemia (Cohort 1: 2 (8.3%); Cohort 2: 1 (4.5%), and nausea (Cohort 1: 3 (12.5%); Cohort 2: 0). Overall, hematological adverse events were infrequent, with the most common hematologic adverse events being anemia (50%) and lymphopenia (30.4%). The most frequent non-hematologic adverse events were nausea (58.7%), fatigue (56.5%), and vomiting (37.0%).

### Treatment efficacy

#### Cohort 1

**Figure 2:**
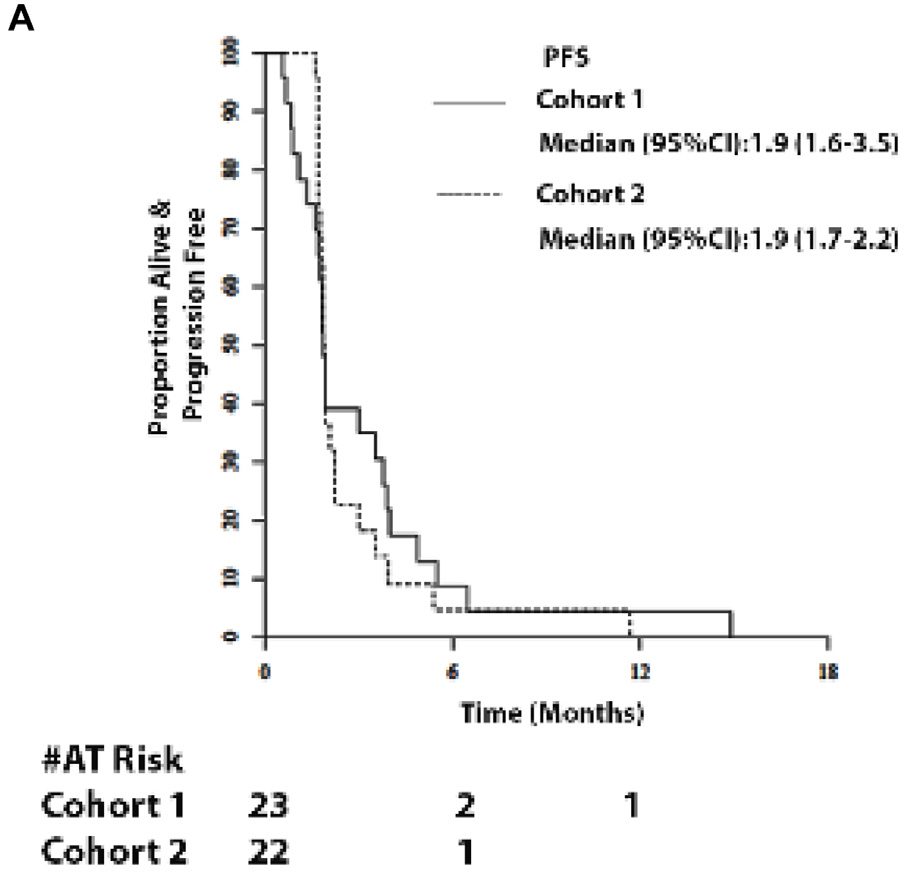
Survival. 2A and 2B. Progression-free survival (PFS) of patients and OS of patients in Cohort 1 and 2

Amongst 23 evaluable patients in Cohort 1, median follow-up was 5.8 months (range 1 - 38.9). No RECIST criteria tumor responses were observed. Median PFS was 1.9 months (95% CI = 1.6 -3.5; Figure 1A) and median OS was 5.6 months (95% CI = 3.0 - 7.7; Figure 2A). The six-month survival rate was 0.5 (95% CI = 0.3 - 0.7). Three patients had stable disease on therapy at the second restaging at month 4 and beyond.

#### Cohort 2

Amongst 22 evaluable patients in Cohort 2, median follow-up was 7.5 months (range 1.8-35.6). No RECIST criteria tumor responses were observed. Median PFS was 1.9 months (95% CI = 1.7 - 2.2; Figure 2A) and median OS was 8.3 months (95% CI = 5.8-12.8; Figure 2B). The six-month survival rate was 0.7 (95% CI = 0.5 - 0.9). One patient had stable disease on therapy at the second restaging at month 4 and beyond.

### DNA methylation analyses

#### Biopsy feasibility

Study-specific biopsies were obtained in 37 patients (82%) at baseline and 19 on treatment at cycle 2 (42%). Of the 19 paired biopsies obtained, 18 had acceptable quality DNA for DNA methylation characterization.

#### DNA demethylation with treatment and association with clinical endpoints

**Figure 3:**
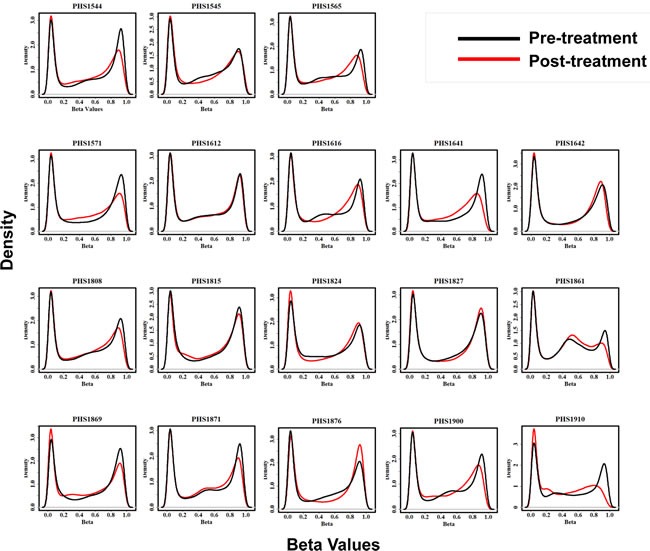
3A Global methylation levels in pre and post treatment samples. Each density plot depicts the distribution of methylation levels across all probes in each of 18 patients before and after treatment as indicated by black and red curves respectively. Blue asterisks (*) indicate thirteen samples that show decrease in methylation level of hypermethylated probes post-treatment. The X-axis represents the beta values while the Y-axis represents methylation density. **3B**. Unsupervised hierarchical clustering of patients based on changes in promoter CpG Island methylation post-treatment and their association with survival. Patient samples are subjected to unsupervised clustering (without clinical annotation) based on their overall change in promoter CpG Island methylation after treatment. Dendrogram at the top reflects similarity of patients based on methylation patterns. Nodes of the dendrogram are annotated as (A) Progression free survival (PFS) above or below the median or (B) Overall survival beyond or less than 6 months; patients alive and lost-to-follow-up before 6 months were censored and coded as NA Subset of patients with similar survival rates show similar change in methylation patterns. Heatmaps depict the delta β values (post β- pre β) at each of the 6219 promoter CpG Island probes in consideration for eighteen patients. Green and red colors in the heatmap represent decrease and increase in methylation respectively in the post-treatment compared to pre-treatment samples. Black represents small or no change.

Genome-wide DNA methylation distributions of each of the 18 paired pre and post-treatment samples is illustrated in Figure 3. The plots exhibit an expected distribution of b-values with distinctive spikes in density of hypomethylated (0 < β-value < 0.2) and hypermethylated (0.8 < β-value < 1) probes. Interestingly, a majority of samples (*n* = 13, 72%), indicated by blue asterisks, exhibit a decrease in methylation levels of hypermethylated probes after treatment. Conversely, only 8 (44%) samples exhibit an increase in methylation levels of hypomethylated probes post treatment.

**Figure 4:**
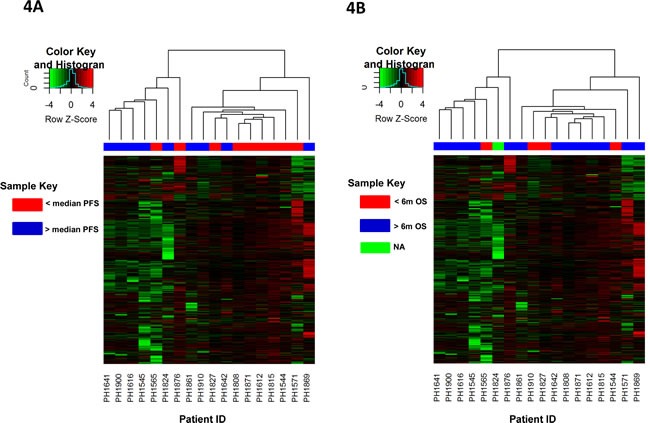
Unsupervised hierarchical clustering of patients based on changes in promoter CpG Island methylation post-treatment and their association with survival Patient samples are subjected to unsupervised clustering (without clinical annotation) based on their overall change in promoter CpG Island methylation after treatment. Dendrogram at the top reflects similarity of patients based on methylation patterns. Nodes of the dendrogram are annotated as **A**. Progression free survival (PFS) above or below the median or **B**. Overall survival beyond or less than 6 months; patients alive and lost-to-follow-up before 6 months were censored and coded as NA Subset of patients with similar survival rates show similar change in methylation patterns. Heatmaps depict the delta β values (post β- pre β) at each of the 6219 promoter CpG Island probes in consideration for eighteen patients. Green and red colors in the heatmap represent decrease and increase in methylation respectively in the post-treatment compared to pre-treatment samples. Black represents small or no change.

Unsupervised clustering of patient samples based on change in DNA methylation of promoter CpG Islands after treatment point to two distinct groups as indicated by the dendrogram (Figure 4). Sample annotation post-clustering indicates a possible association of change in DNA methylation with progression-free survival status in at least a subset of patients, though not overall survival (Supplementary Figure 1A and 1B). Patients with better survival rates show distinctive DNA demethylation for a considerable number of promoter CpG Island probes. Figure 4A groups patients based on PFS above or below the median, while Figure 4B groups patients based on OS beyond or less than 6 months (one patient was alive and lost to follow up before 6 months as indicated by NA).

Statistical analyses pointed to a number of promoter CpG island probes that were differentially methylated between higher and lower survival times. Based on PFS, 213 probes were found to be differentially methylated between patients with low and high survival times at a level of statistical significance (p≤ 0.05); Supplemental Table 1 lists the genes associated with these probes. However, multiple testing correction resulted in a high false discovery rate (FDR) or q-value of 0.976 for the 213 probes. Similarly, 181 probes were differentially methylated based on OS with a p-value cut-off of 0.05 but with a FDR of 1.

We assessed the association of global and promoter demethylation levels with patient survival (Supplementary Table 2). Using Cox proportion hazards models, we analyzed the correlation between promoter and global demethylation percentage with PFS and OS. No significant association was found between promoter demethylation and patient progression or survival [PFS: HR = 0.23, 95%CI:0.03-1.56, *p* = 0.11; OS: HR = 1.21, 95%CI:0.27-5.45, *p* = 0.8 (Table 3)].

**Table 3:** Hypermethylation association with survival

		Hazard Ratio	95% CI^1^	*P* value^2^
Global %	PFS	0.96	(0.85, 1.08)	0.47
	OS	1.02	(0.93, 1.12)	0.66
Promoter %	PFS	0.23	(0.03, 1.56)	0.11
	OS	1.21	(0.27, 5.45)	0.8

^1^ CI: Confidence Interval

^2^ *p*-values are from the Likelihood Ratio Tests

## DISCUSSION

Our study failed to meet its primary RECIST criteria tumor response rate endpoint, with rare patients that had prolonged stable disease up to 10 months. These data corroborate previously published phase I studies evaluating combinations of DNMT inhibitors and HDAC inhibitors in advanced solid tumors and lung cancer and the study of this combination in advanced breast cancer reported concurrently to this study. The totality of these studies suggest that this combination of epigenetic therapy has minimal, if any efficacy in these solid tumors [19, 20–21].

We must measure these results against the efficacy of these agents seen in hematological malignancies, from myelodysplasia and acute leukemias (DNMT inhibitors) to cutaneous lymphomas [HDAC inhibitors [12–15, 22]. Malignant hematologic conditions may be more heavily regulated by epigenetic abnormalities at baseline, and thus more susceptible to an epigenetic therapeutic strategy. Work from our group and others suggest this is not the case; solid tumors including CRC display widespread epigenetic dysregulation with functional consequences including inhibition of cellular differentiation, tumor suppressor gene silencing including as p16 and p53, and regulators of pro-survival pathways such as Wnt/B-catenin, as just a few examples (2-5).

A logical explanation for the lack of efficacy of the combination therapy in this study may be differences in tumor cell exposure to the therapy. Malignant hematologic cells, by virtue of their location in the highly vascular bone marrow or the circulation itself, would potentially have significantly greater exposure to systemic agents. Prior studies by Silverman *et al*. showed that, in patients with hematologic malignancies such as MDS, several months of therapy was required before responses were observed [23]. Our correlative studies also suggest that sub-optimal exposure to our study drugs may have occurred in multiple patients. Paired tumor biopsies in 18 patients showed multiple patients with no DNA demethylation and others with only modest magnitude of effect (Figure 3). Interestingly, those patients with greater DNA demethylation were in the group of patients with a PFS greater than the median versus those below the median, in an unsupervised clustering analysis of patients. Still, this potential benefit did not translate into an overall survival difference between these two groups, and there was no correlation of degree of demethylation with PFS or OS (Supplementary Table 1). We do not have complete data regarding subsequent therapy, which may have an impact on an overall survival endpoint. The patient numbers are small and these analyses are hypothesis-generating in terms of the clinical impact of pharmacodynamics modulation of DNA methylation in mCRC patients with epigenetic agents. The correlative studies on this trial were not planned to assess for changes in histone acetylation, as these changes have not correlated with disease behavior in any previously reported studies of HDAC inhibitors.

Do these agents have any anti-tumor effect and where do we take epigenetic therapy in colorectal cancer in the future from here? We have demonstrated that, upon exposure to these agents, gene expression is increased across many genes, both preclinically in this report, as well as in patients in previously reported work, with epigenetic modulating treatment [24–25]. In particular, our group has reported that azacitidine and entinostat treatment upregulated immune-related genes and create a CpG island methylator phenotype (CIMP)-like gene expression pattern, which correlates with the gene expression seen in microsatellite high (MSI-H) colorectal tumors [11,26]. In a high impact publication, MSI-H colorectal cancer was recently been shown to be highly responsive to immune checkpoint inhibitor therapy [27].

A future approach for epigenetic therapy in colorectal cancer emerges when we combine these data with results from a phase I/II study of advanced non-small cell lung cancer (NSCLC), where patients had improved tumor response rates to immune checkpoint inhibitors as well as cytotoxic chemotherapy after entinostat and azacitidine administration [19]. Using epigenetic agents as priming therapy for both cytotoxic agents and immunotherapy is being explored in multiple tumor types. In NSCLC, azacitidine and entinostat are being evaluated as priming agents for the anti-PD1 agent nivolumab (NCT01928576). We are enrolling a similar strategy of romidepsin (HDAC inhibitor), cc-486 (oral azacitidine) or both with anti-PD1 pembrolizumab in microsatellite stable colorectal cancer. Zhou *et al*. published work showing entinostat resulted in synergistic tumor reduction in *in vivo* models when combined with combination immune checkpoint inhibitors against PD1 and CTLA4; a clinical trial of that triplet is now enrolling (NCT024553620) [28].

Epigenetic agents are also being tested in solid tumors to reverse chemoresistance or prime for increased sensitivity to cytotoxic and hormonal chemotherapy, with trials in ovarian, breast, lung, and colon cancer presently accruing. Matei *et al*. showed a 35% response rate in platinum-resistant ovarian cancer and 6-month PFS of 53% with a combination of decitabine followed by carboplatin [29]. In heavily pretreated NSCLC, 21% of patients responded to subsequent therapies after treatment with entinostat and azacitidine [19]. In a randomized phase II trial of exemestane with or without entinostat in advanced breast cancer patients progressing on hormonal therapy, overall survival was increased significantly by 8.3 months [30]; these findings are now being further tested in a phase III study. In CRC, we are presently enrolling a randomized phase II trial of SGI-110 and irinotecan, in irinotecan-pretreated patients after recently reported the phase I study suggesting possible activity of that combination [31].

Our data suggest that using epigenetic therapy with DNMT and HDAC inhibitor combinations alone may have no activity in colorectal cancer. Novel epigenetic agents such as bromodomain inhibitors and lysine methyltransferase inhibitors are now entering the clinic and may have potential of single-agent activity [32–33]. Until then, we also await the results of ongoing clinical trials of these agents in combination with traditional chemotherapeutic drugs and immunomodulatory agents to hopefully uncover a possible therapeutic benefit of epigenetic agents in CRC.

## MATERIALS AND METHODS

### Colorectal cancer cell lines

Human colorectal cell lines (SW480, SW620, HCT116, and RKO) were acquired from American Type Culture Collection (ATCC) and cultivated and maintained under recommended conditions. Cell lines were treated with varying doses of 5-azacitidine (AZA: up to 500 nM) for three days and/or entinostat (MS-275) up to 1 µM for one day; in the combination treatment group, entinostat was given for 24 hours after the third day of azacitidine treatment was completed. In brief, cells were plated 24 hours prior to the addition of drugs. After 24 hours, drug(s) was added and allowed to incubate for 3 days, while changing the media and drug every 24 hours for AZA treatment. DNA and RNA were obtained by standard protocols from the above cell lines, and were used for Illumina Infinium HumanMethylation450 (HM450) DNA methylation and Agilent 44K Expression array analyses [34].

### Gene expression and methylation array

SW480 and SW620 cell lines were analyzed for gene expression profiles using Agilent Human 4×44K expression arrays (Agilent Technologies, Santa Clara, CA, USA), while DNA methylation analysis was conducted using the HM450 BeadChip (Illumina, Inc., San Diego, CA, USA). Data were processed using R and Bioconductor-based software tools (PMID: 25633503, 15461798).

### Methylation-specific polymerase chain reaction (MSP)

The DNA methylation status of four candidate genes previously reported to be methylated in CRC (*GATA4*, *GATA5*, *SFRP1*, and *TFPI2*) was tested by MSP [35]. Primer sequences used in this study were published previously [36]. DNA was extracted using standard protocol. Bisulfite conversion was carried out using EZ DNA Methylation kit (Zymo Research Corporation) as recommended by the manufacturer. PCR was performed in a 25 µl reaction volume containing 0.5 µl of *Platinum Taq* DNA polymerase (Invitrogen), 2 µl of template DNA and 35 cycles were used at 57°C annealing temperature. *In vitro* methylated DNA (IVD) as a positive control for DNA methylation and HCT116 (*DNMT1*/*DNMT3B* knockout; DKO) DNA as a negative control for DNA methylation were used as positive and negative controls, respectively. Amplification products were analyzed using agarose gel electrophoresis using GelStar™, Stain (Cambrex Bio Science).

### Quantitative real-time polymerase chain reaction (qRT-PCR)

Total RNA for the aforementioned genes was extracted from the above mentioned cell lines. cDNA was synthesized from a total of 1µg of total RNA using random primers and superscript III reverse transcriptase as previously described [37]. Three independent cDNA samples were quantified in triplicate by real-time RT-PCR (PCR conditions initial denaturation 95°C for 5 min, denaturation at 95°C for 30 sec, annealing at 55°C for 30 sec, and finally extension step at 72°C for 30 sec for 40 cycles) using the SYBR green PCR mix (BioRad). mRNA expression was determined using following primer sequences (5’ to 3’):: *GATA4* (F: CTGGCCTGTCATCTCACTACG, R: GGTCCGTGCAGGAATTTGAGG), *GATA5* (F: TCGCCAGCACTGACAGCTCAG, R: TGGTCTGTTCCAGGCTGTTCC), *SFRP1* (F: CCAGCGAGTACGACTACGTGAGCTT, R: CTCAGATTTCAACTCGTTGTCACAGG), and *TFPI-2* (F: GGGCCCTACTTCTCCGTTAC). Gene expression data were normalized to *ACTB* expression (F: TTCTACAATGAGCTGCGTGTG and R: GGGGTGTTGAAGGTCTCAAA).

### Patients

Patients were eligible for the trial if they had histologically confirmed metastatic colorectal cancer, any number of prior therapies, able to undergo tumor biopsy, and measurable disease by Response Evaluation Criteria in Solid Tumors (RECIST) version 1.0, Eastern Cooperative Oncology Group performance status < 1, and adequate hepatic, renal, and hematologic function. Exclusion criteria included chemotherapy within four weeks and previous exposure to HDAC inhibitors or DNMT inhibitors. Institutional review boards of the participating centers approved this protocol. All patients gave written informed consent.

After enrollment of the first 24 patients on study, the study was halted for safety analyses due to crossing pre-specified boundaries of grade 3 toxicities in the first 9 patients. The study was then amended to allow no more than 2 or 3 prior systemic therapies in the metastatic setting (KRAS mutated and KRAS wild type) and limit liver disease burden to < 30% with centralized imaging review to confirm prior to enrollment. The remainder of the eligibility criteria remained constant, and a new cohort was enrolled with modified statistical considerations. The first 24 patients are labeled as Cohort 1, and the next 23 patients in the second group are labeled as Cohort 2.

### Study design

This open-label, single-arm study was opened through the Mayo-Clinic Phase 2 Consortium and sponsored by the National Cancer Institute (NCI) Cancer Therapeutics Evaluation Program (CTEP). The primary objective was to evaluate the efficacy of subcutaneous 5-azacitidine and entinostat in pretreated, metastatic colorectal cancer. The primary endpoint was confirmed tumor response rate, defined as the percentage of patients who show a complete response (CR) or partial response (PR) as the objective status on two consecutive evaluations at least four weeks apart. Secondary endpoints included time to progression, safety and toxicity assessments, and correlative science studies.

Enrolled patients were treated with subcutaneous 5-azacitidine 40 mg/m^2^ days 1-5 and days 8-10 combined with oral entinostat (7 mg flat dose) on days 3 and 10 for 28 day cycles based on the previously reported NSCLC trial [19]. Patients were treated until disease progression or unacceptable adverse events. A maximum of four weeks was permitted for treatment delays due to toxicity.

### Assessments

Patients were evaluated for toxicity each day of the treatment schedule according to the National Cancer Institute Common Toxicity Criteria, version 4.0 (NCI-CTC v4.0). Radiographic tumor response was evaluated every 8 weeks with radiographic scans that were reviewed at each site according to RECIST.

### Correlative analysis

Translational explorations included changes in global DNA gene methylation as well as correlation of these events with clinical outcomes. Tumor biopsies were performed at baseline as well as after two cycles of therapy. Peripheral blood was obtained at baseline, cycle 1 day 3 and 10, and day 1 of each subsequent cycle. Tissue samples were evaluated for DNA demethylation and analyzed using the Illumina Infinium HM450 DNA methylation BeadChip (Illumina).

### Statistical considerations for clinical trial

Cohort 1 was designed as a two-stage, phase II study that required a total of 37 patients to discriminate between true confirmed response rate of ≤5% and ≥20% at a type I error of 9% and power 90%. If one or more patients of the first 12 patients had a confirmed CR (complete response) or PR (partial response), an additional 25 patients would be enrolled. If 4 or more of 37 patients had a confirmed PR or CR, this combination would be considered to have activity in this patient population and would merit further clinical study.

Cohort 2 was also designed as a two-stage, phase II study that required a total of 37 patients to discriminate between true confirmed response rate of ≤5% and ≥20% at a type I error of 9% and power 90%. In this cohort, if 2 or more patients of the first 20 patients had a confirmed CR or PR, an additional 17 patients would be enrolled. Again, if 4 or more of 37 patients had a confirmed PR or CR, this combination would be considered to have activity in this patient population and would merit further clinical study.

Progression-free survival (PFS) and overall survival (OS) were defined from start of therapy to progression of disease and death of any cause, respectively, or last date of follow-up and were analyzed using Kaplan-Meier methods. Patients lost to follow-up were censored. Response rates were calculated with 95% exact confidence intervals (CI). Toxicities were summarized using NCI-CTC v3.0, and the maximum grade per patient was used as the summary measure.

### Statistical considerations for preclinical cell line studies

All data were analyzed using R: A Language and Environment for Statistical Computing [38]. Expression normalization of cell line data was performed using the *limma* R package as previously described [39]. Pathways enriched with a false discovery rate (FDR) less than 0.25 and a normalized enrichment score (NES) >2.15 (upregulated gene sets), or < -2.15 (downregulated gene sets) were chosen. These criteria represented the top ˜30% of all upregulated gene sets as determined by the NES score. Genes were defined as demethylated if they met the following criteria: 1) a high basal β-value (fraction methylated compared to normal) > 0.5 and a ∆β_(AZA-Mock)_ < -0.25, 2) expressed at low basal levels in the untreated cells (lower than the median of the entire group) AND 3) expressed at higher levels in the AZA-treated cells (>2-fold from untreated). The only HM450 probes included in the analysis were those that recognized promoter CpG islands. Demethylated/re-expressed genes were required to meet both DNA demethylation and re-expression criteria in at least one cell line.

### Statistical considerations for clinical correlative studies

Raw files obtained from processing of DNA samples using Illumina's Infinium HM450 array were analyzed using R Bioconductor Package Minfi (v.1.11) [38]. Data were preprocessed using subset quantile within array normalization (SWAN) method. Density plots for β-value distribution depicting global DNA methylation levels were generated from all probes in the array without any filtering with β values in the x-axis and density in the y-axis.

Promoter CpG Island probes were identified from the annotation provided by Illumina. For promoter methylation analysis, only those with β-value higher than 0.4 in at least one of the samples were considered, resulting in 6219 probes. ∆β values (β_Post_-β_Pre_) for the selected probes were used for performing an unsupervised hierarchical clustering using Heatmap.2 package in R using Euclidean distance measure. Values were scaled by rows. Sample annotation based on survival (above or below median PFS or above or below 6 month OS) were included in the dendrogram post-clustering.

The ∆β-values for 6219 promoter probes were subjected to Wilcoxon rank sum test to identify probes that were differentially methylated at a level of statistical significance between each of two groupings of patients based on whether or not they do well in the PFS or OS metric. False discovery rates (FDRs, i.e., q-values) were calculated due to the large number of tests [40–41].

Overall DNA demethylation was assessed globally (genome-wide) and within promoter regions. For calculating global and promoter DNA demethylation percentage for each patient, probes with a delta β cut-off of 0.2 were considered. Additionally any probes belonging to X and Y chromosomes were also filtered out. Percentage of DNA demethylation was calculated by dividing number of probes fulfilling the above criteria by total number of probes expressed as a percentage. Association between PFS and OS and global and promoter DNA demethylation was analyzed using cox proportional hazard model.

## SUPPLEMENTARY MATERIALS FIGURE AND TABLES



## References

[1] Herman JG, Ballin SB (2003). Gene silencing in cancer in association with promoter hypermethylation. N Engl J Med.

[2] Merlo A, Herman JG, Mao L, Lee DJ, Gabrielson E, Burger PC, Baylin SB, Sidransky D (1995). 5′ CpG island methylation is associated with transcriptional silencing of the tumour suppressor p16/CDKN2/MTS1 in human cancers. Nat Med.

[3] Hiltunen MO, Alhonen L, Koistinaho J, Myöhänen S, Pääkkönen M, Marin S, Kosma VM, Jänne J (1997). Hypermethylation of the APC (adenomatous polyposis coli) gene promoter region in human colorectal carcinoma. Int J Cancer.

[4] Caldwell GM, Jones C, Gensberg K, Jan S, Hardy RG, Byrd P, Chughtai S, Wallis Y, Matthews GM, Morton DG (2004). The Wnt antagonist sFRP1 in colorectal tumorigenesis. Cancer Res.

[5] Kane MF, Loda M, Gaida GM, Lipman J, Mishra R, Goldman H, Jessup JM, Kolodner R (1997). Methylation of the hMLH1 promoter correlates with lack of expression of hMLH1 in sporadic colon tumors and mismatch repair-defective human tumor cell lines. Cancer Res.

[6] Yi JM, Dhir M, Van Neste L, Downing SR, Jeschke J, Glöckner SC, de Freitas Calmon M, Hooker CM, Funes JM, Boshoff C, Smits KM, van Engeland M, Weijenberg MP (2011). Genomic and epigenomic integration identifies a prognostic signature in colon cancer. Clin Can Res.

[7] Schuebel KE, Chen W, Cope L, Glöckner SC, Suzuki H, Yi JM, Chan TA, Van Neste L, Van Criekinge W, van den Bosch S, van Engeland M, Ting AH, Jair K (2007). Comparing the DNA hypermethylome with gene mutations in human colorectal cancer. PLoS Genet.

[8] Glöckner SC, Dhir M, Yi JM, McGarvey KE, Van Neste L, Louwagie J, Chan TA, Kleeberger W, de Bruïne AP, Smits KM, Khalid-de Bakker CA, Jonkers DM, Stockbrügger RW (2009). Methylation of TFPI2 in stool DNA: a potential novel biomarker for the detection of colorectal cancer. Cancer Res.

[9] Eads CA, Nickel AE, Laird PW (2002). Complete genetic suppression of polyp formation and reduction of CpG-island hypermethylation in Apc(Min/+) Dnmt1-hypomorphic Mice. Cancer Res.

[10] Toyota M, Ahuja N, Ohe-Toyota M, Herman JG, Baylin SB, Issa JP (1999). CpG island methylator phenotype in colorectal cancer. Proc Natl Acad Sci U S A.

[11] Weisenberger DJ, Siegmund KD, Campan M, Young J, Long TI, Faasse MA, Kang GH, Widschwendter M, Weener D, Buchanan D, Koh H, Simms L, Barker M (2006). CpG island methylator phenotype underlies sporadic microsatellite instability and is tightly associated with BRAF mutation in colorectal cancer. Nat Genet.

[12] Marks P, Rifkind RA, Richon VM, Breslow R, Miller T, Kelly WK (2001). Histone deacetylases and cancer: causes and therapies. Nat Rev Cancer.

[13] Piekarz RL, Frye R, Turner M, Wright JJ, Allen SL, Kirschbaum MH, Zain J, Prince HM, Leonard JP, Geskin LJ, Reeder C, Joske D, Figg WD (2009). Phase II multi-institutional trial of the histone deacetylase inhibitor romidepsin as monotherapy for patients with cutaneous T-cell lymphoma. J Clin Oncol.

[14] Whittaker SJ, Demierre MF, Kim EJ, Rook AH, Lerner A, Duvic M, Scarisbrick J, Reddy S, Robak T, Becker JC, Samtsov A, McCulloch W, Kim YH (2010). Final results from a multicenter, international, pivotal study of romidepsin in refractory cutaneous T-cell lymphoma. J Clin Oncol.

[15] Fenaux P, Mufti GJ, Hellstrom-Lindberg E, Santini V, Finelli C, Giagounidis A, Schoch R, Gattermann N, Sanz G, List A, Gore SD, Seymour JF, Bennett JM (2009). International Vidaza High-Risk MDS Survival Study Group. Efficacy of azacitidine compared with that of conventional care regimens in the treatment of higher-risk myelodysplastic syndromes: a randomised, open-label, phase III study. Lancet Oncol.

[16] Karpf BC, Moore TO, Jones Ririe DA (2001). Activation of the p53 DNA damage response pathway after inhibition of DNA methyltransferase by 5-aza-2-deoxycytidine. Mol Pharmacol.

[17] Palii SS, Van Emburgh BO, Sankpal UT, Brown KD, Robertson KD (2008). DNA methylation inhibitor 5-Aza-2-deoxycytidine induces reversible genome-wide DNA damage that is distinctly influenced by DNA methyltransferases 1 and 3B. Mol Cell Biol.

[18] Cameron EE, Bachman KE, Myöhänen S, Herman JG, Baylin SB (1999). Synergy of demethylation and histone deacetylase inhibition in the re-expression of genes silenced in cancer. Nat Genet.

[19] Juergens RA, Wrangle J, Vendetti FP, Murphy SC, Zhao M, Coleman B, Sebree R, Rodgers K, Hooker CM, Franco N, Lee B, Tsai S, Delgado IE (2011). Combination epigenetic therapy has efficacy in patients with refractory advanced non-small cell lung cancer. Cancer Discov.

[20] Lin J, Gilbert J, Rudek MA, Zwiebel JA, Gore S, Jiemjit A, Zhao M, Baker SD, Ambinder RF, Herman JG, Donehower RC, Carducci MA (2009). A phase I dose-finding study of 5-azacytidine in combination with sodium phenylbutyrate in patients with refractory solid tumors. Clin Cancer Res.

[21] Connolly RM, Jankowitz RC, Zahnow CA, Zhang Z, Rudek MA, Slater S, Powers P, Jeter S, Brufsky A, Piekarz R, Herman JG, Ahuja N, Somlo G (2014). Phase 2 Study Investigating the Safety, Efficacy and Surrogate Biomarkers of Response of 5-Azacitidine (5-AZA) and Entinostat (MS-275) in Advanced Breast Cancer. J Clin Oncol.

[22] Gore SD, Baylin S, Sugar E, Carraway H, Miller CB, Carducci M, Grever M, Galm O, Dauses T, Karp JE, Rudek MA, Zhao M, Smith BD (2006). Combined DNA methyltransferase and histone deacetylase inhibition in the treatment of myeloid neoplasms. Cancer Res.

[23] Silverman LR, McKenzie DR, Peterson BL, Holland JF, Backstrom JT, Beach CL, Larson RA, Cancer Leukemia Group B (2006). Further analysis of trials with azacitidine in patients with myelodysplastic syndrome: studies 8421, 8921, and 9221 by the Cancer and Leukemia Group B. J Clin Oncol.

[24] Li H, Chiappinelli KB, Guzzetta AA, Easwaran H, Yen RW, Vatapalli R, Topper MJ, Luo J, Connolly RM, Azad NS, Stearns V, Pardoll DM, Davidson N (2014). Immune regulation by low doses of the DNA methyltransferase inhibitor 5-azacitidine in common human epithelial cancers. Oncotarget.

[25] Tellez CS, Grimes MJ, Picchi MA, Liu Y, March TH, Reed MD, Oganesian A, Taverna P, Belinsky SA (2014). SGI-110 and entinostat therapy reduces lung tumor burden and reprograms the epigenome. Int J Cancer.

[26] Berg M, Nordgaard O, Kørner H, Oltedal S, Smaaland R, Søreide JA, Søreide K (2015). Molecular subtypes in stage II-III colon cancer defined by genomic instability: early recurrence-risk associated with a high copy-number variation and loss of RUNX3 and CDKN2A. PLoS One.

[27] Le DT Uram JN, Wang H, Bartlett BR, Kemberling H, Eyring AD, Skora AD, Luber BS, Azad NS, Laheru D, Biedrzycki B, Donehower RC, Zaheer A (2015). PD-1 Blockade in Tumors with Mismatch-Repair Deficiency. N Engl J Med.

[28] Kim K, Skora AD, Li Z, Liu Q, Tam AJ, Blosser RL, Diaz LA, Papadopoulos N, Kinzler KW, Vogelstein B, Zhou S (2014). Eradication of metastatic mouse cancers resistant to immune checkpoint blockade by suppression of myeloid-derived cells. Proc Natl Acad Sci U S A.

[29] Matei D, Fang F, Shen C, Schilder J, Arnold A, Zeng Y, Berry WA, Huang T, Nephew KP (2012). Epigenetic resensitization to platinum in ovarian cancer. Cancer Res.

[30] Yardley DA, Ismail-Khan RR, Melichar B, Lichinitser M, Munster PN, Klein PM, Cruickshank S, Miller KD, Lee MJ, Trepel JB (2013). Randomized phase II, double-blind, placebo-controlled study of exemestane with or without entinostat in postmenopausal women with locally recurrent or metastatic estrogen receptor-positive breast cancer progressing on treatment with a nonsteroidal aromatase inhibitor. J Clin Oncol.

[31] Azad N, Zahnow CA, Rudin CM, Baylin SB (2013). The future of epigenetic therapy in solid tumours--lessons from the past. Nat Rev Clin Oncol.

[32] Lee V, Wang J, El Khoueiry A, Verheul H, Gootjes E, Sharma A, Kerner Z, Jones P, Baylin S, Lilly E, Ahuja N, Brown T, Azad NS (2016). A phase I study of guadecitabine (GUA) combined with irinotecan (IRI) in previously treated metastatic colorectal cancer (mCRC) patients. Abstract CT017.

[33] O’Dwyer PJ, Piha-Paul SA, French C, Harward S, Ferron-Brady G, Wu Y, Barbash O, Wyce A, Annan M, Horner T, Parr NJ, Prinja RK, Carpenter C (2016). GSK525762, a selective bromodomain (BRD) and extra terminal protein (BET) inhibitor: results from part 1 of a phase I/II open-label single-agent study in patients with NUT midline carcinoma (NMC) and other cancers. Abstract CT014.

[34] Tsai HC, Li H, Van Neste L, Cai Y, Robert C, Rassool FV, Shin JJ, Harbom KM, Beaty R, Pappou E, Harris J, Yen RW, Ahuja N (2012). Transient low doses of DNA-demethylating agents exert durable antitumor effects on hematological and epithelial tumor cells. Cancer Cell.

[35] Herman JG, Graff JR, Myöhänen S, Nelkin BD, Baylin SB (1996). Methylation-specificPCR: a novel PCR assay for methylation status of CpG islands. Proc Natl Acad Sci U S A.

[36] Dhir M, Yachida S, Van Neste L, Glöckner SC, Jeschke J, Pappou EP, Montgomery EA, Herman JG, Baylin SB, Iacobuzio-Donahue C, Ahuja N (2011). Sessile serrated adenomas and classical adenomas: an epigenetic perspective on premalignant neoplastic lesions of the gastrointestinal tract. Int J Cancer.

[37] Zhang W, Glöckner SC, Guo M, Machida EO, Wang DH, Easwaran H, Van Neste L, Herman JG, Schuebel KE, Watkins DN, Ahuja N, Baylin SB (2008). Epigenetic inactivation of the canonical Wnt antagonist SRY-box containing gene 17 in colorectal cancer. Cancer Res.

[38] R Foundation for Statistical Computing http://www.R-project.org/.

[39] Smyth GK, Michaud J, Scott HS (2005). Use of within-array replicate spots for assessing differential expression in microarray experiments. Bioinformatics.

[40] Goeman JJ, Solari A (2014). Multiple hypothesis testing in genomics. Stat Med.

[41] Storey JD, Tibshirani R (2003). Statistical significance for genomewide studies. Proceedings of the National Academy of Sciences (PNAS).

