# Supplementary material for: Combination epigenetic therapy in metastatic colorectal cancer (mCRC) with subcutaneous 5-azacitidine and entinostat: a phase 2 consortium/stand Up 2 cancer study
Source: Oncotarget. 2017 Feb 5;8(21):35326–38. doi: 10.18632/oncotarget.15108 (PMC5471058; doi:10.18632/oncotarget.15108)
Supplement: Supplementary file 1 [file oncotarget-08-35326-s001.pdf]

Combination epigenetic therapy in metastatic colorectal cancer (mCRC) with subcutaneous 5-azacitidine and entinostat: a phase 2 consortium/stand Up 2 cancer study

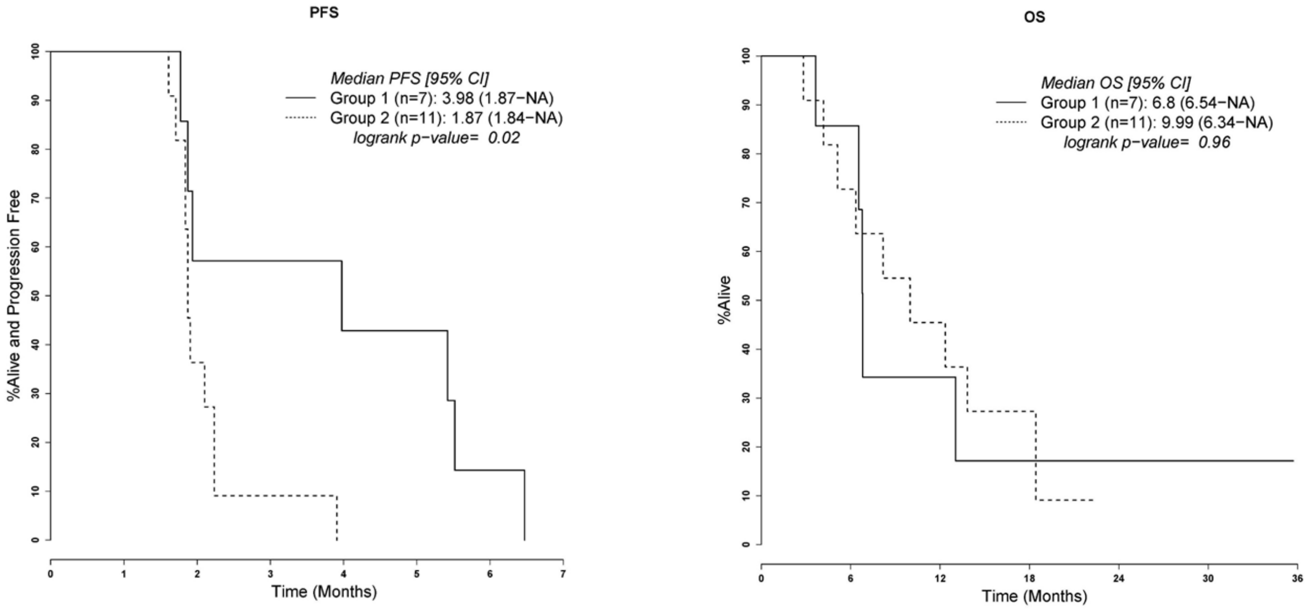

Supplementary Figure 1: Survival grouped by global DNA demethylation in post- versus pre-treatment biopsies. Kaplan-Meier curves were generated for PFS (A) and OS (B) for patients based on global DNA methylation changes with treatment. Group 1 represents patients who had significant global DNA demethylation versus Group 2 patients who did not. Patients with global DNA demethylation had improved PFS, but not OS.

Supplementary Table 1

Differentially methylated genes in > versus < median PFS patients			
ACTN3;ZDHHC24	DEDD2	NSDHL;CETN2	STX1B
ADNP	DHH	OSTC	TAZ;DNASE1L1
AIFM1	DNM1P35	PDLIM2	TECR;MIR639
ALG13	EBP	PDZD11;KIF4A	THOC2
APEX2	FADS2	PHF6	TIMM17B;PQBP1
ARHGEF9	FAM104B	PIK3R5	TMEM164
ATG4A;PSMD10	FCRLB	PJA1	TMEM187;HCFC1
ATP6AP1	FGR	PPP1R3F	TSC22D3
ATP6V0C	FOXO4	PRKACB	TYMP;SCO2
ATP7A	GFI	PSMD10;ATG4A	UCK1
ATRN	GK	PSME1;	UNC45A
B3GAT2	GLA;HNRNPH2	RAB9A	UPF3B
B3GNT7	GNAS	RBBP7	USP4;C3orf62
BCYRN1;INGX	GRB10	RBM20	USP51
BEX4	HLA-L	RBMX;SNORD61	VAMP5
BZRAP1	IGF1R	RNF185	WBP2NL
C10orf125	IGF2BP1	ROPN1L	WDR45
C11orf2	INGX;BCYRN1	RRP15	XIAP
C17orf50	IRGQ;ZNF576	RSU1	ZBTB24
C17orf64	IRS2	RTEL1	ZC3HAV1L
C1orf194;KIAA1324	ITPR2	RWDD2B	ZFP36L1
C3orf67	JOSD1	SCMH1	ZMYM3;BCYRN1
C4orf39;TRIM61	KIF4A;PDZD11	SELK	ZNF138
CACNA1C	L3MBTL	SEPT6	ZNF182;SPACA5
CD320	LMO2	SGCE;PEG10	ZNF275
CD72	LOC100133957;UXT	SLC25A33	ZNF287
CDC25B	LOC91316	SLC35A2	ZNF345
CLCN5	MAP3K7IP3	SMCR8;TOP3A	ZNF502
COL9A2	MECP2;MECP2	SMS	ZNF547;TRAPPC2P1
CORIN	MED27	SNX32	ZNF75D;ZNF449
CXorf58;APOO	MGMT	SPACA5;ZNF182	ZNF81
DCPS	MTCP1;MTCP1NB;BRCC3	SPAG16	
DDX26B	MYADM	SPIN4	
DECR2	MYST4	STAG2	

Supplementary Table 2

Sample ID	Global Demethylation (%)	Promoter Demethylation (%)
PH1544	7.63	0.20
PH1545*#	5.75	0.32
PH1565	8.47	0.72
PH1571#	12.68	1.07
PH1612#	0.70	0.05
PH1616*#	4.59	0.59
PH1641*#	9.23	0.63
PH1642*#	1.37	0.17
PH1808*#	0.22	0.02
PH1815*#	0.04	0.005
PH1824*	8.91	0.90
PH1827	1.31	0.26
PH1861*#	2.69	0.18
PH1869*#	16.21	0.94
PH1871#	0.36	0.02
PH1876#	4.39	0.22
PH1900*#	4.87	0.49
PH1910*	14.42	0.54

*PFS >Median, #OS >6 Months
